# Cumulative Corticosteroid Dose Over Fifty‐Two Weeks in Patients With Systemic Lupus Erythematosus: Pooled Analyses From the Phase III Belimumab Trials

**DOI:** 10.1002/art.39682

**Published:** 2016-08-25

**Authors:** Ronald F. van Vollenhoven, Michelle Petri, Daniel J. Wallace, David A. Roth, Charles T. Molta, Anne E. Hammer, Yongqiang Tang, April Thompson

**Affiliations:** ^1^ Academic Medical CenterAmsterdamThe Netherlands; ^2^ Johns Hopkins University School of MedicineBaltimoreMaryland; ^3^ Cedars–Sinai Medical Center and University of California, Los Angeles David Geffen School of MedicineLos Angeles; ^4^ GlaxoSmithKline, Research Triangle ParkNorth Carolina; ^5^ GlaxoSmithKlinePhiladelphiaPennsylvania

## Abstract

**Objective:**

To examine the effects of treatment with belimumab on corticosteroid dose in patients with systemic lupus erythematosus (SLE) over 52 weeks in 2 randomized, controlled trials.

**Methods:**

Data on patients who were taking corticosteroids at baseline in the Study of Belimumab in Subjects with SLE trials were pooled post hoc to compare patients who received belimumab 10 mg/kg plus standard therapy with those who received placebo plus standard therapy. The primary end point was cumulative change from baseline in corticosteroid dose (prednisone equivalent) through week 52. Further analyses specifically examined oral corticosteroid dose.

**Results:**

At baseline, 966 of 1,125 patients (86%) were receiving corticosteroids (478 belimumab 10 mg/kg and 488 placebo). Most were women (94%), their mean age was 37.1 years, mean Safety of Estrogens in Lupus Erythematosus National Assessment version of the SLE Disease Activity Index score was 9.8, and mean corticosteroid dosage was 12.5 mg/day. Over 52 weeks, there was a smaller increase in mean cumulative corticosteroid dose for the belimumab group than for the placebo group (531.2 mg versus 916.3 mg; *P* < 0.0001). Compared with placebo, the mean of all decreases in cumulative corticosteroid dose was higher with belimumab (*P* = 0.0165), and the mean of all increases was lower (*P* = 0.0005). More patients in the belimumab group had decreases in oral corticosteroid dose (38.5% versus 30.9%), and fewer had increases in dose (18.4% versus 30.7%), compared with placebo. Adverse events were comparable across groups.

**Conclusion:**

Our findings show a significantly smaller increase in cumulative corticosteroid dose over 1 year, more patients with decreases in oral corticosteroid dose, and fewer patients with increases in oral corticosteroid dose in the belimumab group compared with the placebo group. These data suggest that belimumab may be steroid sparing.

The long‐term prognosis of systemic lupus erythematosus (SLE) remains unsatisfactory 1, 2, 3, 4. Long‐term consequences of the disease and its treatment continue to accumulate in many patients, and significant increases in various comorbidities, such as cardiovascular disease, are often seen 5, 6, 7. It is not completely clear what proportion of long‐term complications is due to SLE itself and what proportion is a consequence of continuing exposure to SLE medications, most notably corticosteroids; most experts believe that both contribute 7. A number of studies have demonstrated that corticosteroid use is a key factor in organ damage accrual in patients with SLE, and the risk of organ damage increases with increasing corticosteroid dose 8, 9, 10, 11, 12, 13. Thus, one of the major goals of SLE therapy is to reduce overall cumulative corticosteroid exposure, and it has been suggested that treatment regimens should be evaluated for “corticosteroid‐sparing” effects 14, 15.

Belimumab is a monoclonal antibody directed against B lymphocyte stimulator 16. In 2 large randomized, controlled trials, the Study of Belimumab in Subjects with SLE 52‐week (BLISS‐52) and 76‐week (BLISS‐76) trials, belimumab plus standard therapy reduced SLE disease activity compared with standard therapy alone (placebo) 17, 18. These trials featured a pragmatic design whereby background medications could be adjusted to some extent, based on the patient's condition. Thus, during several periods of the trial, corticosteroid doses could be increased if the patient experienced worsening symptoms, and decreased if the symptoms were reduced. This flexibility in the BLISS‐52 and BLISS‐76 trial designs supported a post hoc analysis of the combined data set to examine the overall effect of increases or decreases in corticosteroid dose, and whether there was a difference between patients who received belimumab and those who received placebo. We anticipated an overall reduction in cumulative or average daily corticosteroid exposure, and hypothesized that belimumab would permit a greater reduction than placebo.

## PATIENTS AND METHODS

### Study design

Data from the BLISS trials were pooled post hoc (GSK200317). Patients who were randomized to receive belimumab 10 mg/kg (the licensed dose) plus standard therapy or placebo plus standard therapy were included (n = 1,125), and patients who were treated with corticosteroids at baseline were analyzed. The study design and findings of the BLISS‐52 trial (n = 865) (ClinicalTrials.gov identifier: NCT00424476; GSK110751) and BLISS‐76 trial (n = 819) (ClinicalTrials.gov identifier: NCT00410384; GSK110752) have been reported previously 17, 18. Briefly, patients were randomized to receive belimumab 1 mg/kg or 10 mg/kg plus standard therapy or placebo plus standard therapy by intravenous (IV) infusion on days 0, 14, and 28, and then every 28 days until week 48 (BLISS‐52) or week 72 (BLISS‐76). Changes in corticosteroid dose were permitted during the first 24 weeks of the BLISS trials. Increases in corticosteroid dose (above baseline or week 44 dose) were not allowed during weeks 44–52. During weeks 24–52, treatment was deemed to have failed if the total systemic corticosteroid dose was >25% or 5 mg higher than the baseline corticosteroid dose (whichever was higher).

In this post hoc analysis, changes from baseline in cumulative corticosteroid dose were compared over 52 weeks. Baseline dose was defined as the average corticosteroid dose (prednisone equivalent for all oral, IV, subcutaneous [SC], or intramuscular [IM] administrations) over the 7 days prior to, but not including, day 0; this was used to model the normalized cumulative baseline dose over 52 weeks. Actual cumulative corticosteroid dose (prednisone equivalent for all oral, IV, SC, or IM administrations) over 52 weeks was calculated. In the primary analysis, to calculate the cumulative change over the remaining study period for patients who withdrew prior to week 52, last corticosteroid dose was calculated by imputing the average corticosteroid dose over the 7 days (primary method of imputation) prior to withdrawal. Two sensitivity analyses were conducted for withdrawals to examine the possible impact of pulse steroid administration: imputing the average corticosteroid dose over the previous 28 days among patients who had completed ≥8 weeks of treatment, and imputing the average dose for the entire treatment period that the patient was in the study.

### Study end points

The primary end point was the net cumulative change from baseline in corticosteroid dose, defined as the net change from baseline in daily corticosteroid dose (all routes of administration) from day 0 to day 364. Secondary end points included cumulative decrease in corticosteroid dose, defined as the sum of change from baseline (area under the curve [AUC]) in daily corticosteroid dose on days the dose was less than baseline (from day 0 to day 364); cumulative increase in corticosteroid dose, defined as the sum of change from baseline (AUC) in daily corticosteroid dose on days the dose was greater than baseline (from day 0 to day 364); and change from baseline in average daily corticosteroid dose, defined as the net cumulative change from baseline in corticosteroid dose divided by the total number of follow‐up days. Average daily increase or decrease in corticosteroid dose over the study period (defined as cumulative increase or decrease in corticosteroid dose divided by the total number of follow‐up days) and the percentage of patients who had an increase or decrease in dose were exploratory end points. In further post hoc analyses, changes in corticosteroid dose were also calculated among patients who had an SLE Responder Index (SRI) response during the BLISS trials, and changes in body weight, blood pressure (BP), and glucose levels for those patients in the highest and lowest quartiles for cumulative corticosteroid dose were examined.

The primary population for analysis included all patients who received corticosteroids at baseline. In addition, a subgroup with high disease activity, defined as patients with serologically active disease who were anti–double‐stranded DNA (anti‐dsDNA) positive and had low complement levels at baseline, was analyzed. This serologic definition of high disease activity is associated with more severe disease, and therefore this population is of particular interest 19, 20.

All end points were analyzed in further post hoc analyses that included only oral corticosteroid doses. To capture patients who withdrew prior to week 52, oral corticosteroid dose was calculated by imputing the average dose over the 7 days (primary method of imputation) prior to withdrawal. A sensitivity analysis was also conducted, imputing the entire treatment period for patients who withdrew.

Adverse events (AEs) were summarized by treatment group and according to the highest and lowest quartiles for cumulative corticosteroid dose (for all routes of administration and for oral corticosteroids only [post hoc analysis]).

### Statistical analysis

The original BLISS trials were not designed to examine corticosteroid management and were not powered to examine changes in corticosteroid dose; this post hoc analysis was conducted to assess trends in cumulative corticosteroid dose changes over 52 weeks. Rank analysis of covariance with a 2‐sided alpha level of 0.05 was applied to corticosteroid dose end points to compare treatment groups. Covariates in the model included treatment group, baseline prednisone dosage (mg/day), baseline Safety of Estrogens in Lupus Erythematosus National Assessment (SELENA) version of the SLE Disease Activity Index (SLEDAI) score (≤9 versus ≥10), baseline proteinuria level (<2 gm/24 hours versus ≥2 gm/24 hours equivalent), and race (African descent or American Indian descent versus other). No correction for multiple comparisons was conducted.

## RESULTS

### Patient population

At baseline, 86% of the study population (966 of 1,125 patients) received corticosteroid therapy; 478 of these patients were randomized to receive belimumab 10 mg/kg plus standard therapy and 488 to receive placebo plus standard therapy. Baseline demographic and clinical characteristics for patients receiving corticosteroids were similar between treatment groups (Table 1). Overall, the majority were women (94%), their mean age was 37.1 years, and the mean corticosteroid dosage (prednisone equivalent) was 12.5 mg/day. The mean SELENA–SLEDAI score was 9.8, and 71.5% of the patients were anti‐dsDNA positive. There were 537 patients with high disease activity (275 in the group treated with belimumab 10 mg/kg and 262 in the group treated with placebo).

**Table 1:** Baseline demographic and clinical characteristics of patients treated with corticosteroids at baseline in the BLISS‐52 and BLISS‐76 trials^a^

	Belimumab 10 mg/kg (n = 478)	Placebo (n = 488)	Total (n = 966)
Sex, female/male	457 (95.6)/21 (4.4)	453 (92.8)/35 (7.2)	910 (94.2)/56 (5.8)
Age, mean ± SD years	36.8 ± 11.0	37.3 ± 12.0	37.1 ± 11.5
BMI, mean ± SD kg/m^2^	25.0 ± 5.6	25.2 ± 5.3	25.1 ± 5.4
SELENA–SLEDAI score, mean ± SD	9.9 ± 3.8	9.7 ± 3.8	9.8 ± 3.8
PGA score, mean ± SD	1.4 ± 0.5	1.5 ± 0.5	1.4 ± 0.5
Proteinuria level, mean ± SD^b^	0.5 ± 0.9	0.5 ± 1.1	0.5 ± 1.0
BILAG organ domain involvement			
At least 1A or 2B	280 (58.6)	299 (61.3)	579 (59.9)
At least 1A	72 (15.1)	82 (16.8)	154 (15.9)
At least 1A or 1B	431 (90.2)	447 (91.6)	878 (90.9)
No A or B	47 (9.8)	41 (8.4)	88 (9.1)
At least 1 SLE flare	83 (17.4)	104 (21.3)	187 (19.4)
SLICC damage index score, mean ± SD	0.7 ± 1.2	0.7 ± 1.2	0.7 ± 1.2
ANA positive (titer ≥80)^c^	447 (93.5)	453 (92.8)	900 (93.2)
Anti‐dsDNA positive (≥30 IU/ml)	353 (73.8)	338 (69.3)	691 (71.5)
Low C3 (<900 mg/liter)^d^	242 (50.6)	227 (46.5)	469 (48.6)
Low C4 (<16 mg/dl)	295 (61.7)	269 (55.1)	564 (58.4)
Corticosteroid dose, mean ± SD	12.8 ± 8.5	12.3 ± 7.9	12.5 ± 8.2

a Except where indicated otherwise, values are the number (%) of patients. BLISS‐52 = Study of Belimumab in Subjects with SLE 52‐week trial; BMI = body mass index; SELENA–SLEDAI = Safety of Estrogens in Lupus Erythematosus National Assessment version of the Systemic Lupus Erythematosus Disease Activity Index; PGA = physician's global assessment; BILAG = British Isles Lupus Assessment Group; SLICC = Systemic Lupus International Collaborating Clinics; ANA = antinuclear antibody.

b Twenty‐four–hour equivalent as measured by spot urine protein‐to‐creatinine ratio.

c Maximum titer of the individual patterns.

d A high disease activity subgroup included 537 patients who were anti–double‐stranded DNA (anti‐dsDNA) positive and had low C3 and C4 levels (275 were receiving belimumab 10 mg/kg and 262 were receiving placebo).

The percentage of patients who withdrew from the study was higher in the group treated with placebo than in the group treated with belimumab 10 mg/kg. In the placebo‐treated group, 7.6% of the patients (37 of 488) withdrew before week 24, and 17.5% of the patients (79 of 451) withdrew at week 24 or later. In the belimumab‐treated group, 6.3% of the patients (30 of 478) withdrew before week 24 and 13.4% of the patients (60 of 448) withdrew at week 24 or later.

### Route of administration of corticosteroids

During the study the percentage of patients receiving IV or parenteral steroids was similar between treatment groups. In the group receiving placebo, 11.1% (55 of 488) received IV steroids, 5.1% (25 of 488) received parenteral steroids, and 15.0% (73 of 488) received both IV and parenteral steroids. In the group receiving belimumab 10 mg/kg, 11.6% (56 of 478) received IV steroids, 3.7% (18 of 478) received parenteral steroids, and 14.6% (70 of 478) received both IV and parenteral steroids.

### Cumulative change in corticosteroid dose

**Figure 1:**
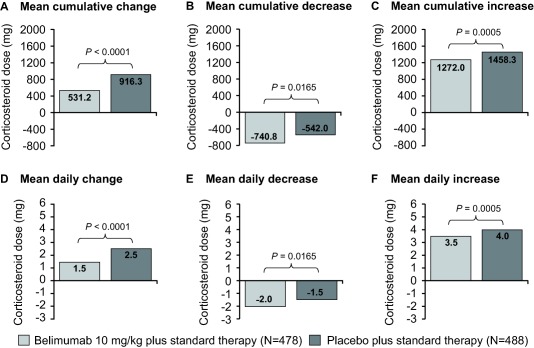
Change in corticosteroid dose (all routes of administration) for patients treated with belimumab 10 mg/kg and patients treated with placebo. **A–C,** Mean cumulative change from baseline in corticosteroid dose over 52 weeks. Cumulative decrease was defined as the area under the curve (AUC) for days on which the change from baseline was <0. Cumulative increase was defined as the AUC for days on which the change from baseline was >0. **D–F,** Mean change in daily corticosteroid dose over 52 weeks (7‐day imputation). Daily change was defined as cumulative change from baseline in corticosteroid dose divided by the total number of follow‐up days. All doses were normalized based on the number of days the patient was in the study.

The overall mean cumulative change in corticosteroid dose from baseline to 52 weeks (primary end point) was 531 mg in the group receiving belimumab 10 mg/kg compared with 916 mg in the group receiving placebo (*P* < 0.0001) (Figure 1A). This smaller overall increase in the belimumab‐treated group occurred as the mean of all cumulative decreases in corticosteroid dose was greater in the belimumab‐treated group than in the placebo‐treated group, while the mean of all cumulative increases was greater in the placebo‐treated group than in the belimumab‐treated group (Figures 1B and C).

The sensitivity analysis using the average corticosteroid dose over the previous 28 days (among patients who had completed ≥8 weeks of the study) showed results similar to the primary end point; the overall mean cumulative change was 599 mg in the belimumab 10 mg/kg–treated group compared with 923 mg in the placebo‐treated group (*P =* 0.0124). However, when the entire treatment period was imputed for patients who withdrew, the overall change was 645 mg for belimumab 10 mg/kg compared with 616 mg for placebo (*P =* 0.0180).

Supplementary Figure 1A, available on the *Arthritis & Rheumatology* web site at http://onlinelibrary.wiley.com/doi/10.1002/art.39682/abstract, illustrates that changes in cumulative steroid dose between the 30th and 70th percentiles were similar between the patients treated with belimumab 10 mg/kg and those treated with placebo. However, above the 70th percentile, increases in cumulative steroid doses appeared generally greater for the placebo‐treated group than for the belimumab 10 mg/kg–treated group.

The overall mean cumulative change from baseline was 1,015 mg for belimumab compared with 1,560 mg for placebo (*P =* 0.0015) in the high disease activity subgroup (Supplementary Figure 2, available on the *Arthritis & Rheumatology* web site at http://onlinelibrary.wiley.com/doi/10.1002/art.39682/abstract).

### Change in daily corticosteroid dose

The mean overall change in daily corticosteroid dose from baseline to 52 weeks was 1.5 mg for patients receiving belimumab 10 mg/kg and 2.5 mg for patients receiving placebo (*P* < 0.0001) (Figure 1D). As with the cumulative dose, this smaller overall increase in daily dose with belimumab was brought about by a greater mean daily decrease and a smaller mean daily increase with belimumab than with placebo (Figures 1E and F). Similar results were observed in the high disease activity subgroup (mean overall change 2.8 mg/day versus 4.3 mg/day; *P* = 0.0015) (Supplementary Figure 2).

An overall increase in corticosteroid dose over 52 weeks was observed in 22.6% of the patients who received belimumab 10 mg/kg compared with 35.0% of the patients who received placebo. Conversely, a decrease in corticosteroid dose was observed in a greater proportion of patients who received belimumab 10 mg/kg compared with patients who received placebo (37.0% versus 29.7%).

### Change in oral corticosteroid dose

**Figure 2:**
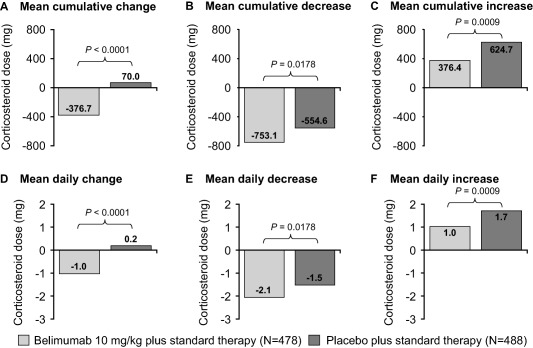
Change in oral corticosteroid dose for patients treated with belimumab 10 mg/kg and patients treated with placebo. **A–C,** Mean cumulative change from baseline in oral corticosteroid dose over 52 weeks. Cumulative decrease was defined as the area under the curve (AUC) for days on which the change from baseline was <0. Cumulative increase was defined as the AUC for days on which the change from baseline was >0. **D–F,** Mean change in daily oral corticosteroid dose over 52 weeks (7‐day imputation). Daily change was defined as cumulative change from baseline in oral corticosteroid dose divided by the total number of follow‐up days. All doses were normalized based on the number of days the patient was in the study.

There was an overall mean cumulative decrease from baseline in oral corticosteroid dose of 376.7 mg for the belimumab 10 mg/kg–treated group compared with an increase of 70.0 mg for the placebo‐treated group (*P* < 0.0001) (Figures 2A–C). In the sensitivity analysis, the overall mean cumulative dose increased by 301 mg for patients receiving belimumab 10 mg/kg compared with 427 mg for patients receiving placebo (*P* = 0.0108) when imputing the entire study period for patients who withdrew (average dose for the entire treatment period that the patient remained in the study was imputed). The mean decrease from baseline in daily oral corticosteroid dose was 1.0 mg for the patients receiving belimumab compared with an increase of 0.2 mg for the patients receiving placebo (*P* < 0.0001) (Figures 2D–F).

An overall increase in oral corticosteroid dose over 52 weeks was observed in 18.4% of the patients who received belimumab 10 mg/kg compared with 30.7% of the patients who received placebo. Conversely, a decrease in oral corticosteroid dose was observed in more patients who received belimumab 10 mg/kg than in patients who received placebo (38.5% versus 30.9%).

Supplementary Figure 1B shows that when only oral corticosteroids were considered, the differences between the treatment groups when patients were ranked according to cumulative change in dose were more apparent than when all corticosteroids were included.

### Cumulative change in corticosteroid dose among SRI responders

Among SRI responders, there was a greater decrease in the mean cumulative change from baseline in total corticosteroid dose for the patients treated with belimumab 10 mg/kg (decrease of 696.5 mg; n = 254) compared with the patients treated with placebo (decrease of 367.2 mg; n = 189) (*P* = 0.0076). For oral corticosteroids only, the decrease was also greater for the patients treated with belimumab 10 mg/kg (decrease of 725.5 mg; n = 254) compared with those treated with placebo (decrease of 408.5 mg; n = 189) (*P* = 0.0041).

### Changes in weight, BP, and glucose levels for patients in the highest and lowest cumulative corticosteroid dose quartiles

There was a steady weight gain over 52 weeks for patients in the highest quartile of cumulative corticosteroid dose (mean ± SD change from baseline at week 52 2.5 ± 4.66 kg for patients receiving belimumab [n = 79] and 2.5 ± 6.71 kg for patients receiving placebo [n = 79]), with less weight gain in the lowest quartile (Table 2). Similarly, weight changes appeared greater in patients in the highest quartile of cumulative oral corticosteroid dose compared with patients in the lowest quartile.

**Table 2:** Change in weight, BP, and glucose levels from baseline to week 52 in patients in the highest and lowest cumulative corticosteroid dose quartiles^a^

	Highest quartile of cumulative corticosteroid dose		Lowest quartile of cumulative corticosteroid dose		Highest quartile of cumulative oral corticosteroid dose		Lowest quartile of cumulative oral corticosteroid dose	
	Belimumab 10 mg/kg (n = 79)	Placebo (n = 80)	Belimumab 10 mg/kg (n = 112)	Placebo (n = 99)	Belimumab 10 mg/kg (n = 99)	Placebo (n = 90)	Belimumab 10 mg/kg (n = 112)	Placebo (n = 97)
Weight, kg	2.5 ± 4.66	2.5 ± 6.71^b^	0.8 ± 4.08	−0.1 ± 3.56	2.5 ± 4.50	2.3 ± 6.26^b^	0.7 ± 4.19	0.0 ± 3.42
Systolic BP, mm Hg	−2.2 ± 17.62	−2.8 ± 17.20	−3.2 ± 15.96	−3.6 ± 14.10	−2.9 ± 16.89	−2.3 ± 16.52	−3.4 ± 16.01^b^	−3.3 ± 14.62
Diastolic BP, mm Hg	−2.5 ± 11.52	−2.5 ± 12.77	−1.5 ± 10.60	−1.9 ± 10.38	−2.2 ± 10.77	−2.4 ± 12.31	−1.3 ± 10.80^b^	−1.6 ± 10.45
Glucose, mmoles/liter	0.3 ± 1.90	0.1 ± 1.44^b^	0.2 ± 1.46	0.0 ± 1.41^b^	0.2 ± 1.79	0.1 ± 1.34^b^	0.1 ± 1.45	0.0 ± 1.42^b^

a Values are the mean ± SD. BP = blood pressure.

b Data were missing for 1 patient.

Changes from baseline in systolic and diastolic BP varied over the 52‐week period for patients in the highest quartile of cumulative corticosteroid dose, with an overall decrease at week 52 (Table 2). For patients in the lowest quartile the decrease in systolic BP appeared slightly larger and the decrease in diastolic BP appeared slightly smaller compared with the highest quartile. Similar results were observed for oral corticosteroids only.

Changes in blood glucose levels over 52 weeks appeared similar between patients in the lowest and highest quartiles of cumulative corticosteroid dose and oral corticosteroid dose, and between patients treated with belimumab and those treated with placebo (Table 2).

### Safety

The overall incidence of AEs, serious AEs, and drug‐related AEs was similar across the 2 treatment groups (Table 3). The most common drug‐related AEs in both treatment groups were headache, upper respiratory tract infection, arthralgia, and urinary tract infection. There was a similar incidence of all infection and infestation AEs (69.7% in the belimumab‐treated group and 65.2% in the placebo‐treated group) and those classified as serious (6.1% in the belimumab‐treated group and 5.9% in the placebo‐treated group) in both treatment groups. The overall incidence of AEs, serious AEs, and drug‐related AEs was lower among patients in the lowest quartile of cumulative corticosteroid dose compared with those in the highest quartile of cumulative corticosteroid dose for both treatment groups. However, the difference was generally greater in patients receiving belimumab 10 mg/kg compared with those receiving placebo. The incidence of AEs in the lowest and highest quartiles of cumulative oral corticosteroid dose are shown in Supplementary Table 1, available on the Arthritis & Rheumatology web site at http://onlinelibrary.wiley.com/doi/10.1002/art.39682/abstract.

**Table 3:** Summary of AEs occurring over 52 weeks in patients treated with corticosteroids at baseline in the BLISS‐52 and BLISS‐76 trials^a^

	Belimumab 10 mg/kg			Placebo		
	All (n = 478)	Lowest quartile of cumulative corticosteroid dose (n = 129)	Highest quartile of cumulative corticosteroid dose (n = 113)	All (n = 488)	Lowest quartile of cumulative corticosteroid dose (n = 113)	Highest quartile of cumulative corticosteroid dose (n = 129)
Any AE^b^	440 (92.1)	112 (86.8)	108 (95.6)	448 (91.8)	104 (92.0)	123 (95.3)
Headache	98 (20.5)	22 (17.1)	27 (23.9)	106 (21.7)	20 (17.7)	26 (20.2)
Upper respiratory tract infection	71 (14.9)	18 (14.0)	17 (15.0)	83 (17.0)	18 (15.9)	20 (15.5)
Arthralgia	66 (13.8)	26 (20.2)	11 (9.7)	64 (13.1)	15 (13.3)	19 (14.7)
Urinary tract infection	58 (12.1)	11 (8.5)	17 (15.0)	54 (11.1)	13 (11.5)	15 (11.6)
Diarrhea	56 (11.7)	14 (10.9)	18 (15.9)	36 (7.4)	8 (7.1)	7 (5.4)
Nasopharyngitis	53 (11.1)	14 (10.9)	10 (8.8)	42 (8.6)	17 (15.0)	7 (5.4)
Nausea	49 (10.3)	14 (10.9)	14 (12.4)	48 (9.8)	9 (8.0)	17 (13.2)
Back pain	43 (9.0)	13 (10.1)	13 (11.5)	40 (8.2)	5 (4.4)	13 (10.1)
Pyrexia	43 (9.0)	8 (6.2)	19 (16.8)	37 (7.6)	3 (2.7)	16 (12.4)
Influenza	42 (8.8)	10 (7.8)	12 (10.6)	31 (6.4)	9 (8.0)	7 (5.4)
Bronchitis	39 (8.2)	10 (7.8)	7 (6.2)	19 (3.9)	5 (4.4)	6 (4.7)
Edema peripheral	38 (7.9)	5 (3.9)	17 (15.0)	38 (7.8)	5 (4.4)	16 (12.4)
Cough	37 (7.7)	11 (8.5)	7 (6.2)	36 (7.4)	6 (5.3)	9 (7.0)
Fatigue	32 (6.7)	10 (7.8)	6 (5.3)	31 (6.4)	11 (9.7)	8 (6.2)
Insomnia	31 (6.5)	10 (7.8)	8 (7.1)	23 (4.7)	3 (2.7)	6 (4.7)
Vomiting	30 (6.3)	6 (4.7)	12 (10.6)	26 (5.3)	2 (1.8)	9 (7.0)
Hypertension	29 (6.1)	4 (3.1)	13 (11.5)	47 (9.6)	7 (6.2)	15 (11.6)
Pharyngitis	28 (5.9)	7 (5.4)	4 (3.5)	19 (3.9)	5 (4.4)	4 (3.1)
Abdominal pain	28 (5.9)	7 (5.4)	11 (9.7)	21 (4.3)	4 (3.5)	10 (7.8)
Pain in extremity	28 (5.9)	8 (6.2)	4 (3.5)	16 (3.3)	4 (3.5)	4 (3.1)
Dizziness	27 (5.6)	5 (3.9)	4 (3.5)	30 (6.1)	3 (2.7)	8 (6.2)
Myalgia	25 (5.2)	7 (5.4)	8 (7.1)	31 (6.4)	5 (4.4)	8 (6.2)
Cystitis	25 (5.2)	8 (6.2)	7 (6.2)	16 (3.3)	4 (3.5)	4 (3.1)
Sinusitis	25 (5.2)	9 (7.0)	2 (1.8)	24 (4.9)	5 (4.4)	8 (6.2)
Anemia	25 (5.2)	6 (4.7)	8 (7.1)	28 (5.7)	7 (6.2)	6 (4.7)
Pruritus	24 (5.0)	8 (6.2)	4 (3.5)	22 (4.5)	6 (5.3)	3 (2.3)
Gastroenteritis	23 (4.8)	6 (4.7)	7 (6.2)	20 (4.1)	5 (4.4)	8 (6.2)
Rash	22 (4.6)	7 (5.4)	8 (7.1)	22 (4.5)	6 (5.3)	7 (5.4)
Weight increase	21 (4.4)	4 (3.1)	8 (7.1)	13 (2.7)	0	8 (6.2)
Upper abdominal pain	19 (4.0)	4 (3.1)	6 (5.3)	25 (5.1)	4 (3.5)	7 (5.4)
Arthritis	19 (4.0)	5 (3.9)	3 (2.7)	20 (4.1)	6 (5.3)	4 (3.1)
Gastritis	15 (3.1)	2 (1.6)	4 (3.5)	15 (3.1)	3 (2.7)	7 (5.4)
Mouth ulceration	15 (3.1)	5 (3.9)	3 (2.7)	22 (4.5)	5 (4.4)	7 (5.4)
Noncardiac chest pain	18 (3.8)	8 (6.2)	1 (0.9)	22 (4.5)	3 (2.7)	6 (4.7)
Gastroenteritis viral	13 (2.7)	7 (5.4)	2 (1.8)	6 (1.2)	3 (2.7)	1 (0.8)
Hypokalemia	12 (2.5)	3 (2.3)	6 (5.3)	7 (1.4)	1 (0.9)	2 (1.6)
Lupus nephritis	11 (2.3)	2 (1.6)	3 (2.7)	18 (3.7)	3 (2.7)	8 (6.2)
Oropharyngeal pain	9 (1.9)	2 (1.6)	3 (2.7)	13 (2.7)	6 (5.3)	3 (2.3)
Pneumonia	9 (1.9)	1 (0.8)	4 (3.5)	13 (2.7)	1 (0.9)	7 (5.4)
Dyspnea	6 (1.3)	1 (0.8)	3 (2.7)	22 (4.5)	3 (2.7)	8 (6.2)
Any serious AE	89 (18.6)	16 (12.4)	33 (29.2)	80 (16.4)	12 (10.6)	36 (27.9)
Any study agent–related AE	179 (37.4)	38 (29.5)	49 (43.4)	196 (40.2)	45 (39.8)	52 (40.3)

a Values are the number (%). BLISS‐52 = Study of Belimumab in Subjects with SLE 52‐week trial.

b Adverse events (AEs) occurring in ≥5% of patients in any quartile.

## DISCUSSION

This post hoc analysis of the combined BLISS‐52 and BLISS‐76 data sets demonstrates that, over 52 weeks of treatment, corticosteroid doses were decreased more often, and were increased less often, in the belimumab‐treated group than in the placebo‐treated group. Overall, the mean cumulative dose and the mean daily dose increased for patients in both groups, but to a lesser extent in the belimumab‐treated group than in the placebo‐treated group. However, according to the sensitivity analysis (nonparametric statistical analysis) where the entire treatment period was imputed for those patients who withdrew, the increase in overall mean cumulative change in corticosteroids was greater for the patients treated with belimumab 10 mg/kg than for the patients treated with placebo. This paradoxical result is most likely related to the highly skewed distribution of corticosteroid doses in the population, underscoring that nonparametric statistics are appropriate in this case.

Unexpectedly, overall exposure to all corticosteroids increased on average for both treatment groups during these trials. In retrospect, this is perhaps not surprising. The patient population in these trials had longstanding, moderate to highly active SLE despite ongoing background therapy with antimalarials, immunosuppressive agents, and corticosteroids 17, 18. Such patients are at considerable risk of disease flare, and during the trials flares were commonly treated with increases in corticosteroid dose. Such dose increases would typically be moderate to large in size. In contrast, for patients with positive outcomes during the trial it was possible to reduce the corticosteroid dose, as evidenced by the decrease in doses of both all corticosteroids and oral corticosteroids in SRI responders. However, typical dose reductions would have been cautious and of smaller magnitude than increases; thus, it is apparent that the fewer large increases in doses outweighed the more numerous but much smaller dose reductions during the trials.

Our results underscore the great need for effective corticosteroid‐sparing treatments and strategies in SLE. The cumulative exposures seen in this study are at levels expected to result in considerable long‐term toxicities. Yet, the AE data did not reveal consistent differences indicating toxicities. We speculate that any differences in the long‐term consequences of changes in corticosteroid treatment would require longer follow‐up, beyond 1 year, to become measureable.

Belimumab was associated with a greater likelihood of corticosteroid dose reduction and a greater average reduction, and conversely with a lower likelihood of dose increase and a smaller average increase, for both all corticosteroids and oral corticosteroids only. Thus, the data support the consideration of belimumab as a potential steroid‐sparing medication. The numerical differences were not dramatic, which may be related to the fact that this trial was not designed with the primary goal of examining belimumab for potential steroid‐sparing properties. A forced‐steroid‐taper design such as has been used previously in trials of prasterone 21 and abatacept 22 might more clearly demonstrate corticosteroid‐sparing efficacy for belimumab.

Incidences of AEs were similar across the 2 treatment groups and were consistent with the known safety profile of belimumab.

Limitations to the interpretation of these data include the post hoc nature of the analyses, the fact that corticosteroid dose changes were not prescribed in detail by the protocol, the need for statistical imputations of some data, the mixing of oral and parenteral corticosteroids for some analyses, and the lack of power for detecting uncommon corticosteroid‐related side effects. Strengths of the study include the robust combined‐trial sample size and the detailed documentation of corticosteroid doses at the monthly study visits.

In summary, this combined post hoc analysis of data from the BLISS‐52 and BLISS‐76 clinical trials shows that 1 year of treatment with belimumab plus standard therapy is associated with a reduction in the frequency of corticosteroid dose increases, an increase in the frequency of dose decreases, and favorable differences in the magnitude of these corticosteroid dosing changes compared with standard therapy alone. Taken together, these findings support the hypothesis that belimumab may be a corticosteroid‐sparing agent in the treatment of SLE.

## AUTHOR CONTRIBUTIONS

All authors were involved in drafting the article or revising it critically for important intellectual content, and all authors approved the final version to be published. Dr. van Vollenhoven had full access to all of the data in the study and takes responsibility for the integrity of the data and the accuracy of the data analysis.

**Study conception and design.** Van Vollenhoven, Petri, Wallace, Roth, Molta, Hammer, Tang, Thompson.

**Acquisition of data.** Van Vollenhoven, Wallace, Molta, Tang.

**Analysis and interpretation of data.** Van Vollenhoven, Petri, Wallace, Roth, Molta, Hammer, Tang, Thompson.

## ROLE OF THE STUDY SPONSOR

Human Genome Sciences and GlaxoSmithKline designed and conducted the study in collaboration with all authors. GlaxoSmithKline is committed to publicly disclosing the results of GlaxoSmithKline–sponsored clinical research that evaluates GlaxoSmithKline medicines, and as such was involved in the decision to submit. The authors, interpreted the results and had the final decision to submit the manuscript for publication. Medical writing assistance was provided by Louisa Pettinger, PhD (Fishawack Indicia Ltd) and funded by GlaxoSmithKline. Publication of this article was not contingent upon approval by Human Genome Sciences or GlaxoSmithKline.

## Supporting information

Figure S1. Percent ranking of corticosteroid dose change versus actual change at 52 weeks for (A) all corticosteroids and (B) oral corticosteroids only x‐axis represents each individual patient's percentile ranking in their treatment group.

Figure S2. All corticosteroids, high disease activity population: (A–C) mean cumulative change from baseline in corticosteroid dose (7‐day imputation); (D–F) mean change in daily corticosteroid dose over 52 weeks

Supplementary Table 1. Summary of AEs occurring over 52 weeks in patients in the lowest and highest quartiles of oral corticosteroid dose

Supplementary Figure Legends
