# Supplementary material for: Cumulative Corticosteroid Dose Over Fifty‐Two Weeks in Patients With Systemic Lupus Erythematosus: Pooled Analyses From the Phase III Belimumab Trials
Source: Arthritis Rheumatol. 2016 Aug 25;68(9):2184–92. doi: 10.1002/art.39682 (PMC5129492; doi:10.1002/art.39682)
Supplement: Supplementary file 3 — Supplementary Table 1. Summary of AEs occurring over 52 weeks in patients in the lowest and highest quartiles of oral corticosteroid dose [file ART-68-2184-s003.doc]

Supplementary Table 1. Summary of AEs occurring over 52 weeks in patients in the lowest and highest quartiles of oral corticosteroid dose

|  | **Belimumab  10 mg/kg** | | **Placebo** | |
| --- | --- | --- | --- | --- |
|  | **Cumulative oral corticosteroid dose** | | **Cumulative oral corticosteroid dose** | |
|  | **Lowest quartile (N=129)**  **n (%)** | **Highest quartile (N=133)**  **n (%)** | **Lowest quartile (N=114)**  **n (%)** | **Highest quartile (N=136)**  **n (%)** |
| Any AE^a^ | 112 (86.8) | 106 (79.7) | 105 (92.1) | 114 (83.8) |
| Headache | 23 (17.8) | 26 (19.5) | 20 (17.5) | 25 (18.4) |
| Upper respiratory tract infection | 18 (14.0) | 18 (13.5) | 18 (15.8) | 20 (14.7) |
| Arthralgia | 24 (18.6) | 12 (9.0) | 15 (13.2) | 18 (13.2) |
| Urinary tract infection | 11 (8.5) | 17 (12.8) | 13 (11.4) | 12 (8.8) |
| Diarrhea | 14 (10.9) | 17 (12.8) | 9 (7.9) | 6 (4.4) |
| Nasopharyngitis | 17 (13.2) | 10 (7.5) | 17 (14.9) | 7 (5.1) |
| Nausea | 14 (10.9) | 14 (10.5) | 11 (9.6) | 14 (10.3) |
| Back pain | 12 (9.3) | 12 (9.0) | 5 (4.4) | 13 (9.6) |
| Pyrexia | 8 (6.2) | 17 (12.8) | 4 (3.5) | 13 (9.6) |
| Influenza | 10 (7.8) | 11 (8.3) | 9 (7.9) | 7 (5.1) |
| Bronchitis | 11 (8.5) | 8 (6.0) | 5 (4.4) | 4 (2.9) |
| Edema peripheral | 4 (3.1) | 17 (12.8) | 6 (5.3) | 13 (9.6) |
| Cough | 11 (8.5) | 8 (6.0) | 5 (4.4) | 9 (6.6) |
| Fatigue | 11 (8.5) | 6 (4.5) | 11 (9.6) | 8 (5.9) |
| Insomnia | 11 (8.5) | 7 (5.3) | 3 (2.6) | 6 (4.4) |
| Vomiting | 6 (4.7) | 11 (8.3) | 2 (1.8) | 6 (4.4) |
| Hypertension | 4 (3.1) | 14 (10.5) | 8 (7.0) | 15 (11.0) |
| Pharyngitis | 7 (5.4) | 4 (3.0) | 5 (4.4) | 4 (2.9) |
| Abdominal pain | 6 (4.7) | 11 (8.3) | 4 (3.5) | 8 (5.9) |
| Pain in extremity | 7 (5.4) | 4 (3.0) | 3 (2.6) | 4 (2.9) |
| Dizziness | 5 (3.9) | 4 (3.0) | 5 (4.4) | 7 (5.1) |
| Myalgia | 7 (5.4) | 7 (5.3) | 5 (4.4) | 7 (5.1) |
| Cystitis | 8 (6.2) | 6 (4.5) | 5 (4.4) | 4 (2.9) |
| Sinusitis | 10 (7.8) | 2 (1.5) | 5 (4.4) | 8 (5.9) |
| Anemia | 4 (3.1) | 7 (5.3) | 7 (6.1) | 7 (5.1) |
| Pruritus | 8 (6.2) | 3 (2.3) | 6 (5.3) | 2 (1.5) |
| Gastroenteritis | 5 (3.9) | 8 (6.0) | 5 (4.4) | 8 (5.9) |
| Rash | 7 (5.4) | 8 (6.0) | 6 (5.3) | 6 (4.4) |
| Weight increased | 4 (3.1) | 8 (6.0) | 0 | 7 (5.1) |
| Arthritis | 5 (3.9) | 3 (2.3) | 6 (5.3) | 4 (2.9) |
| Gastritis | 2 (1.6) | 4 (3.0) | 3 (2.6) | 7 (5.1) |
| Mouth ulceration | 4 (3.1) | 1 (0.8) | 4 (3.5) | 8 (5.9) |
| Non-cardiac chest pain | 7 (5.4) | 1 (0.8) | 3 (2.6) | 6 (4.4) |
| Gastroenteritis viral | 7 (5.4) | 2 (1.5) | 3 (2.6) | 0 |
| Lupus nephritis | 2 (1.6) | 3 (2.3) | 4 (3.5) | 8 (5.9) |
| Dyspnea | 0 | 2 (1.5) | 3 (2.6) | 7 (5.1) |
| Any SAE | 17 (13.2) | 31 (23.3) | 15 (13.2) | 29 (21.3) |
| Any study agent-related AE | 39 (30.2) | 50 (37.6) | 45 (39.5) | 46 (33.8) |

^a^AEs occurring in ≥5% of patients in any quartile.
AE, adverse event; SAE, serious adverse event
