# Supplementary material for: Cumulative Corticosteroid Dose Over Fifty‐Two Weeks in Patients With Systemic Lupus Erythematosus: Pooled Analyses From the Phase III Belimumab Trials
Source: Arthritis Rheumatol. 2016 Aug 25;68(9):2184–92. doi: 10.1002/art.39682 (PMC5129492; doi:10.1002/art.39682)
Supplement: Supplementary file 4 — Supplementary Figure Legends [file ART-68-2184-s004.doc]

### Figure S1. Percent ranking of corticosteroid dose change versus actual change at 52 weeks for (A) all corticosteroids and (B) oral corticosteroids only

x-axis represents each individual patient’s percentile ranking in their treatment group.

Figure S2. All corticosteroids, high disease activity population: (A–C) mean cumulative change from baseline in corticosteroid dose (7-day imputation); (D–F) mean change in daily corticosteroid dose over 52 weeks
